# Support Vector Machines and Kernels for Computational Biology

**DOI:** 10.1371/journal.pcbi.1000173

**Published:** 2008-10-31

**Authors:** Asa Ben-Hur, Cheng Soon Ong, Sören Sonnenburg, Bernhard Schölkopf, Gunnar Rätsch

**Affiliations:** 1 Department of Computer Science, Colorado State University, Fort Collins, Colorado, United States of America; 2 Friedrich Miescher Laboratory, Max Planck Society, Tübingen, Germany; 3 Max Planck Institute for Biological Cybernetics, Tübingen, Germany; 4 Fraunhofer Institute FIRST, Berlin, Germany; Whitehead Institute, United States of America

## Introduction

The increasing wealth of biological data coming from a large variety of platforms and the continued development of new high-throughput methods for probing biological systems require increasingly more sophisticated computational approaches. Putting all these data in simple-to-use databases is a first step; but realizing the full potential of the data requires algorithms that automatically extract regularities from the data, which can then lead to biological insight.

Many of the problems in computational biology are in the form of prediction: starting from prediction of a gene's structure, prediction of its function, interactions, and role in disease. Support vector machines (SVMs) and related kernel methods are extremely good at solving such problems [1]–[3]. SVMs are widely used in computational biology due to their high accuracy, their ability to deal with high-dimensional and large datasets, and their flexibility in modeling diverse sources of data [2], [4]–[6].

**Figure 1:**
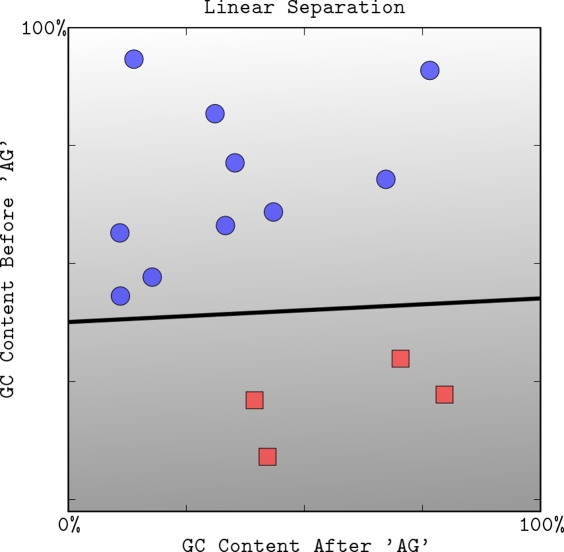
A linear classifier separating two classes of points (squares and circles) in two dimensions. The decision boundary divides the space into two sets depending on the sign of *f*(**x**) = 〈**w,x**〉+*b*. The grayscale level represents the value of the discriminant function *f*(**x**): dark for low values and a light shade for high values.

**Figure 2 pcbi-1000173-g002:**
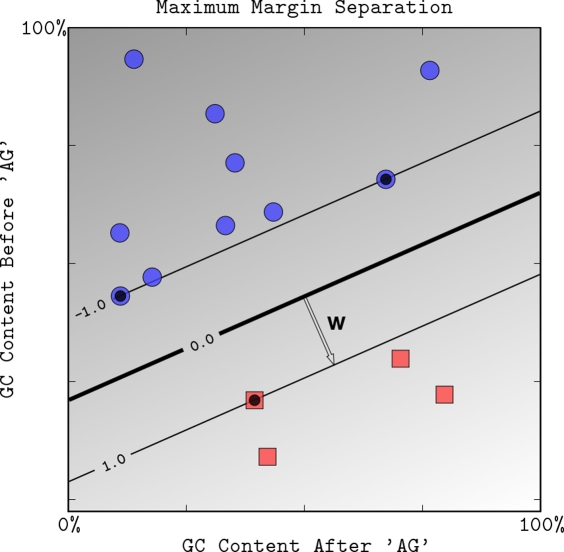
The maximum margin boundary computed by a linear SVM. The region between the two thin lines defines the *margin area* with −1≤〈**w,x**〉+*b*≤1. The data points highlighted with black centers are the *support vectors*: the examples that are closest to the decision boundary. They determine the margin by which the two classes are separated. Here, there are three support vectors on the edge of the margin area (*f*(**x**) = −1 or *f*(**x**) = +1).

**Figure 3 pcbi-1000173-g003:**
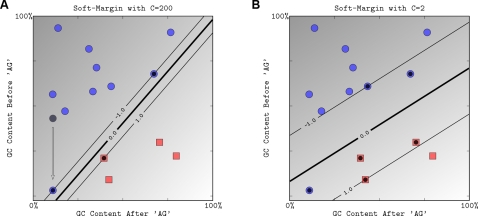
The effect of the soft-margin constant, *C*, on the decision boundary. We modified the toy dataset by moving the point shaded in gray to a new position indicated by an arrow, which significantly reduces the margin with which a hard-margin SVM can separate the data. (A) We show the margin and decision boundary for an SVM with a very high value of *C*, which mimics the behavior of the hard-margin SVM since it implies that the slack variables *ξ_i_* (and hence training mistakes) have very high cost. (B) A smaller value of *C* allows us to ignore points close to the boundary, and increases the margin. The decision boundary between negative examples and positive examples is shown as a thick line. The thin lines are on the margin (discriminant value equal to −1 or +1).

The simplest form of a prediction problem is binary classification: trying to discriminate between objects that belong to one of two categories—positive (+1) or negative (−1). SVMs use two key concepts to solve this problem: large margin separation and kernel functions. The idea of large margin separation can be motivated by classification of points in two dimensions (see Figure 1). A simple way to classify the points is to draw a straight line and call points lying on one side positive and on the other side negative. If the two sets are well separated, one would intuitively draw the separating line such that it is as far as possible away from the points in both sets (see Figures 2 and 3). This intuitive choice captures the idea of *large margin separation*, which is mathematically formulated in the section Classification with Large Margin.

Instead of the abstract idea of points in space, one can think of our data points as representing objects using a set of *features* derived from measurements performed on each object. For instance, in the case of Figures 1–5, there are two measurements for each object, depicted as points in a two-dimensional space. For large margin separation, it turns out that not the exact location but only the relative position or *similarity* of the points to each other is important. In the simplest case of linear classification, the similarity of two objects is computed by the dot-product (a.k.a. scalar or inner product) between the corresponding feature vectors. To define different similarity measures leading to *nonlinear classification* boundaries (cf. Figures 6 and 7), one can extend the idea of dot products between points with the help of *kernel functions* (cf. the section Kernels: From Linear to Nonlinear Classifiers). Kernels compute the similarity of two points and are the second important concept of SVMs and *kernel methods* [2],[7].

**Figure 4 pcbi-1000173-g004:**
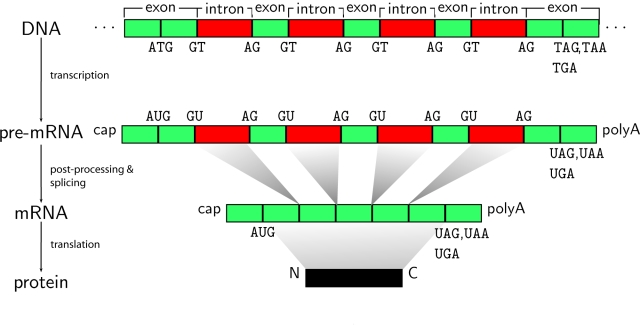
The major steps in protein synthesis: transcription, post-processing, and translation. In the post-processing step, the pre-mRNA is transformed into mRNA. One necessary step in the process of obtaining mature mRNA is called *splicing*. The mRNA sequence of a eukaryotic gene is “interrupted” by noncoding regions called *introns*. A gene starts with an exon and may then be interrupted by an intron, followed by another exon, intron, and so on until it ends in an exon. In the splicing process, the introns are removed. There are two different splice sites: the exon–intron boundary, referred to as the donor site or 5′ site (of the intron), and the intron–exon boundary, that is, the acceptor or 3′ site. Splice sites have quite strong consensus sequences, i.e., almost every position in a small window around the splice site is representative of the most frequently occurring nucleotide when many existing sequences are compared in an alignment (cf. Figure 5). (The caption text appeared similarly in [30], the idea for this figure is from [11].)

**Figure 5 pcbi-1000173-g005:**
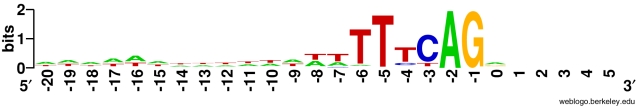
Sequence logo for acceptor splice sites: splice sites have quite strong consensus sequences, i.e., almost every position in a small window around the splice site is representative of the most frequently occurring nucleotide when many existing sequences are compared in an alignment. The sequence logo [72],[73] shows the region around the intron/exon boundary—the acceptor splice site. In the running example, we use the region up to 40 nt upstream and downstream of the consensus site AG.

**Figure 6 pcbi-1000173-g006:**
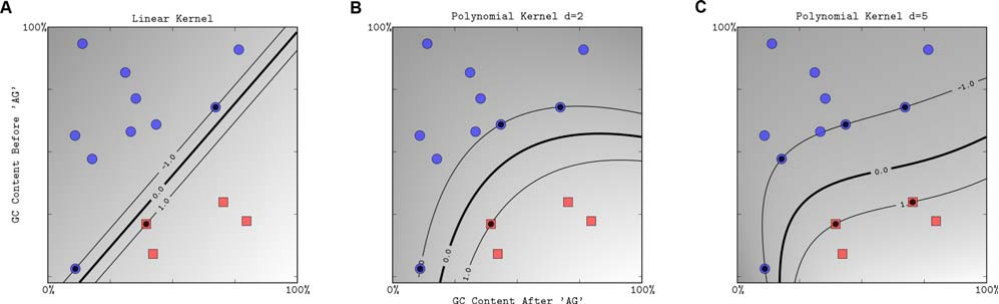
The effect of the degree of a polynomial kernel. The polynomial kernel of degree 1 leads to a linear separation (A). Higher-degree polynomial kernels allow a more flexible decision boundary (B,C). The style follows that of Figure 3.

**Figure 7:**
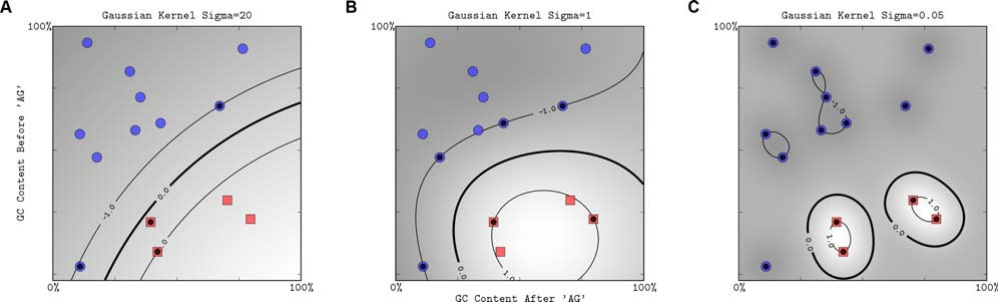
The effect of the width parameter of the Gaussian kernel (*σ*) for a fixed value of the soft-margin constant. For large values of *σ* (A), the decision boundary is nearly linear. As *σ* decreases, the flexibility of the decision boundary increases (B). Small values of *σ* lead to overfitting (C). The figure style follows that of Figure 3.

The domain knowledge inherent in any classification task is captured by defining a suitable kernel (i.e., similarity) between objects. As we shall see later, this has two advantages: the ability to generate nonlinear decision boundaries using methods designed for linear classifiers; and the possibility of applying a classifier to data that have no obvious vector space representation; for example, DNA/RNA, or protein sequences, or protein structures.

### 

#### 

##### Running example: Splice site recognition

Throughout this tutorial we are going to use an example problem for illustration. It is a problem arising in computational gene finding and concerns the recognition of splice sites that mark the boundaries between exons and introns in eukaryotes. Introns are excised from premature mRNAs in a processing step after transcription (see Figure 4 and, for instance, [8]–[12] for more details).

The vast majority of all splice sites are characterized by the presence of specific dimers on the intronic side of the splice site: GT for donor and AG for acceptor sites (see Figure 5). However, only about 0.1%–1% of all GT and AG occurrences in the genome represent true splice sites. In this tutorial, we consider the problem of recognizing acceptor splice sites as a running example, which allows us to illustrate different properties of SVMs using different kernels (similar results can be obtained for donor splice sites as well [13]).

In the first part of the tutorial we are going to use real-valued features describing the sequence surrounding the splice site. For illustration purposes, we use only two features: the GC content in the exon and intron flanking potential *acceptor* sites. These features are motivated by the fact that the GC-content of exons is typically higher than that of introns (see, e.g., Figure 1). In the second part, we show how to take advantage of the flanking pre-mRNA sequence itself, leading to considerable performance improvements. (The data used in the numerical examples was generated by taking a random subset of 200 true splice sites and 2,000 decoy sites from the first 100,000 entries in the *C. elegans* acceptor splice site dataset from [13] (cf. http://www.fml.tuebingen.mpg.de/raetsch/projects/splice). Note that this dataset is much smaller than the original dataset, and is also less unbalanced. In the graphical examples in this tutorial, we show only a small and selected subset of the data suitable for illustration purposes. In practice, there is a considerably stronger overlap in the space of GC content between positive examples (true acceptor sites) and negative examples (other occurrences of AG) than appears on the figures.

To evaluate the classifier performance, we will use so-called *receiver operating characteristic* (ROC) curves [14], which show the true positive rates (*y*-axis) over the full range of false positive rates (*x*-axis). Different values are obtained by using different thresholds on the value of the discriminant function for assigning the class membership. The area under the curve quantifies the quality of the classifier, and a larger value indicates better performance. Research has shown that it is a better measure of classifier performance than the success or error rate of the classifier [15], in particular when the fraction of examples in one class is much smaller than the other. (Please note that the auROC is independent of the class ratios. Hence, its value is not necessarily connected with the success of identifying rare events. The area under the so-called precision recall curve is better suited to evaluate how well one can find rare events [16].)

##### SVM toolbox

All computational results in this tutorial were generated using the *Shogun*-based *Easysvm* tool [17] written in python [18],[19]. The source code to generate the figures and results is provided under the GNU General Public License [20] at http://svmcompbio.tuebingen.mpg.de. That site also provides a Web service that allows one to train and evaluate SVMs. An alternative implementation using PyML is also available [70].

## Large Margin Separation

### 

#### Linear separation with hyperplanes

In this section, we introduce the idea of linear classifiers. Support vector machines are an example of a linear two-class classifier. The data for a two-class learning problem consists of objects labeled with one of two labels; for convenience we assume the labels are +1 (positive examples) and −1 (negative examples). Let **x** denote a vector with *M* components *x_j_*, *j* = 1,…,*M*, i.e., a point in an *M*-dimensional vector space. The notation **x** *_i_* will denote the *i* ^th^ vector in a dataset 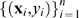, where *y_i_* is the label associated with **x** *_i_*, and n is the number of examples. The objects **x** *_i_* are called *patterns*, *inputs*, and also *examples*.

A key concept required for defining a linear classifier is the *dot product* between two vectors 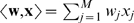, also referred to as the *inner product* or *scalar product*. A linear classifier is based on a linear *discriminant function* of the form
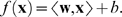
(1)


The discriminant function *f*(**x**) assigns a “score” for the input **x**, and is used to decide how to classify it. The vector **w** is known as the *weight vector*, and the scalar *b* is called the *bias*. In two dimensions, the points satisfying the equation 〈**w**,**x**〉 = 0 correspond to a line through the origin, in three dimensions a plane, and more generally a *hyperplane*. The bias translates the hyperplane with respect to the origin (see Figure 1). (Unlike many schematic representations that the reader may have seen, the figures in this paper are generated by actually applying the SVM on the data points as shown. More details, including code and data, are available at http://svmcompbio.tuebingen.mpg.de.)

The hyperplane divides the space into two half spaces according to the sign of *f*(**x**), that indicates on which side of the hyperplane a point is located (see Figure 1): if *f*(**x**)>0, then one decides for the positive class, otherwise for the negative. The boundary between regions classified as positive and negative is called the *decision boundary* of the classifier. The decision boundary defined by a hyperplane (cf. Equation 1) is said to be *linear* because it is linear in the input. (Note that strictly speaking, for *b*≠0, this is *affine* rather than linear, but we will ignore this distinction.) A classifier with a linear decision boundary is called a linear classifier. In the next section, we introduce one particular linear classifier, the (linear) Support Vector Machine, which turns out to be particularly well suited to high-dimensional data.

#### Classification with large margin

Whenever a dataset such as is shown in Figure 1 is linearly separable, i.e., there exists a hyperplane that correctly classifies all data points, there exist many such separating hyperplanes. We are thus faced with the question of which hyperplane to choose, ensuring that not only the training data, but also *future examples*, unseen by the classifier at training time, are classified correctly. Our intuition as well as statistical learning theory [3] suggest that hyperplane classifiers will work better if the hyperplane not only separates the examples correctly, but does so with a large margin. Here, the margin of a linear classifier is defined as the distance of the closest example to the decision boundary, as shown in Figure 2. Let us adjust *b* such that the hyperplane is half way in between the closest positive and negative examples. If, moreover, we scale the discriminant function, Equation 1, to take the values ±1 for these examples, we find that the margin is 1/∥**w**∥, where ∥**w**∥ is the length of **w**, also known as its *norm*, given by 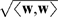 [2].

The so-called *hard margin SVM*, applicable to linearly separable data, is the classifier with maximum margin among all classifiers that correctly classify all the input examples (see Figure 2). To compute **w** and *b* corresponding to the maximum margin hyperplane, one has to solve the following optimization problem:
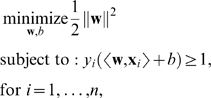
(2)where the constraints ensure that each example is correctly classified, and minimizing ∥**w**∥^2^ is equivalent to maximizing the margin. (The set of formulas above describes a *quadratic optimization problem*, in which the optimal solution (**w**,*b*) is described to satisfy the constraints *y_i_*(〈**w**,**x** *_i_*〉+*b*)≥1, while the length of **w** is as small as possible. Such optimization problems can be solved using standard tools from convex optimization (see, e.g., [21]). For specific optimization problems like the one above, there exist specialized techniques to efficiently solve such optimization problems for millions of examples or dimensions.)

##### Soft margin

In practice, data are often not linearly separable; and even if they are, a greater margin can be achieved by allowing the classifier to misclassify some points—see Figure 3. Theory and experimental results show that the resulting larger margin will generally provide better performance than the hard margin SVM. To allow errors, we replace the inequality constraints in Equation 2 with
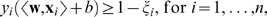
where *ξ_i_*≥0 are *slack variables* that allow an example to be in the margin or misclassified. To discourage excess use of the slack variables, a term *C*Σ_i_ *ξ_i_* is added to the function to be optimized:
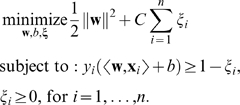
(3)


The constant *C*>0 sets the relative importance of maximizing the margin and minimizing the amount of slack. This formulation is called the *soft-margin SVM* [22].

The effect of the choice of *C* is illustrated in Figure 3. For a large value of *C*, a large penalty is assigned to errors. This is seen in Figure 3A, where the two points closest to the hyperplane strongly affect its orientation, leading to a hyperplane that comes close to several other data points. When *C* is decreased (Figure 3B), those points move inside the margin, and the hyperplane's orientation is changed, leading to a much larger margin for the rest of the data. Note that the scale of *C* has no direct meaning, and there is a formulation of SVMs that uses a more intuitive parameter 0<*ν*≤1 instead. The parameter *ν* controls the fraction of support vectors, and of margin errors (*ν*-SVM, see [2],[7]).

##### Dual formulation

Using the method of Lagrange multipliers (see, e.g., [21]), we can obtain the *dual* formulation. (The dual optimization problem is a reformulation of the original, primal optimization problem. It typically has as many variables as the primal problem has constraints. Its objective value at optimality is equal to the optimal objective value of the primal problem, under certain conditions; see, e.g., [21] for more details.) It is expressed in terms of variables *α_i_* [2],[22]:
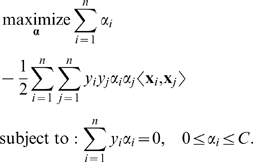
(4)


One can prove that the weight vector **w** in Equation 3 can be expressed in terms of the examples **x** *_i_* and the solution *α_i_* of the above optimization problem as
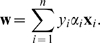
(5)


The **x** *_i_* for which *α_i_*>0 are called *support vectors*; they can be shown to lie on or within the margin (points with black circles in Figures 2–7). Intuitively, all other training examples do not contribute to the geometric location of the large margin hyperplane—the solution would have been the same even if they had not been in the training set to begin with. It is thus not surprising that they drop out of the expansion in Equation 5.

Note that the dual formulation of the SVM optimization problem depends on the inputs **x** *_i_* only through dot products. In the next section, we will show that the same holds true for the discriminant function given by Equation 1. This will allow us to “kernelize” the algorithm.

## Kernels: From Linear to Nonlinear Classifiers

In many applications, a nonlinear classifier provides better accuracy. And yet linear classifiers have advantages, one of them being that they often have simple training algorithms that scale well with the number of examples [23],[24]. This begs the question whether the machinery of linear classifiers can be extended to generate nonlinear decision boundaries. Furthermore, can we handle domains such as biological sequences where a vector space representation is not necessarily available?

There is a straightforward way of turning a linear classifier nonlinear, or making it applicable to nonvectorial data. It consists of mapping our data to some vector space, which we will refer to as the feature space, using a function *φ*. The discriminant function then is
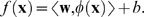
(6)


Note that *f*(**x**) is linear in the feature space defined by the mapping *φ*; but when viewed in the original input space, it is a nonlinear function of **x** if *φ*(**x**) is a nonlinear function. The simplest example of such a mapping is one that considers all products of pairs of features (related to the polynomial kernel; see below). The resulting classifier has a quadratic discriminant function (see example in Figure 6B). This approach of explicitly computing nonlinear features does not scale well with the number of input features. The dimensionality of the feature space associated with the above example is quadratic in the number of dimensions of the input space. If we were to use monomials of degree *d* rather than degree 2 monomials as above, the dimensionality would be exponential in *d*, resulting in a substantial increase in memory usage and the time required to compute the discriminant function. If our data are high-dimensional to begin with, such as in the case of gene expression data, this is not acceptable. Kernel methods avoid this complexity by avoiding the step of explicitly mapping the data to a high-dimensional feature space.

We have seen above (Equation 5) that the weight vector of a large margin separating hyperplane can be expressed as a linear combination of the training points, i.e., 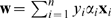. The same holds true for a large class of linear algorithms, as shown by the *representer theorem* (see [2]). Our discriminant function then becomes
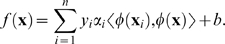
(7)


The representation in terms of the variables *α_i_* is known as the *dual* representation (cf. the section Classification with Large Margin). We observe that the dual representation of the discriminant function depends on the data only through dot products in feature space. The same observation holds for the dual optimization problem (Equation 4) when we replace **x** *_i_* with *φ*(**x** *_i_*) (analogously for **x** *_j_*).

If the *kernel function k*(**x**,**x**′) defined as
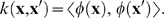
(8)can be computed efficiently, then the dual formulation becomes useful, as it allows us to solve the problem without ever carrying out the mapping *φ* into a potentially very high-dimensional space. The recurring theme in what follows is to define meaningful similarity measures (kernel functions) that can be computed efficiently.

### 

#### Kernels for real-valued data

Real-valued data, i.e., data where the examples are vectors of a given dimensionality, are common in bioinformatics and other areas. A few examples of applying SVM to real-valued data include prediction of disease state from microarray data (see, e.g., [25]), and prediction of protein function from a set of features that include amino acid composition and various properties of the amino acids in the protein (see, e.g., [26]).

The two most commonly used kernel functions for real-valued data are the polynomial and the Gaussian kernel. The *polynomial kernel* of degree *d* is defined as:
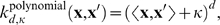
(9)where *κ* is often chosen to be 0 (homogeneous) or 1 (inhomogeneous). The feature space for the inhomogeneous kernel consists of all monomials with degree up to *d* [2]. And yet, its computation time is linear in the dimensionality of the input space. The kernel with *d* = 1 and *κ* = 0, denoted by *k* ^linear^, is the *linear kernel* leading to a linear discriminant function.

The degree of the polynomial kernel controls the flexibility of the resulting classifier (Figure 6). The lowest degree polynomial is the linear kernel, which is not sufficient when a nonlinear relationship between features exists. For the data in Figure 6, a degree 2 polynomial is already flexible enough to discriminate between the two classes with a good margin. The degree 5 polynomial yields a similar decision boundary, with greater curvature. Normalization (cf. the section Normalization) can help to improve performance and numerical stability for large *d*.

The second very widely used kernel is the *Gaussian kernel* defined by
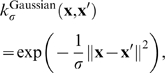
(10)where *σ*>0 is a parameter that controls the width of the Gaussian. It plays a similar role as the degree of the polynomial kernel in controlling the flexibility of the resulting classifier (see Figures 6 and 7). The Gaussian kernel is essentially zero if the squared distance ∥**x**−**x′**∥^2^ is much larger than *σ*; i.e., for a fixed **x′** there is a region around **x′** with high kernel values. The discriminant function (Equation 7) is thus a sum of Gaussian “bumps” centered around each support vector (SV). When *σ* is large (Figure 7A), a given data point **x** has a nonzero kernel value relative to any example in the set of examples. Therefore, the whole set of SVs affects the value of the discriminant function at **x**, leading to a smooth decision boundary. As we decrease *σ*, the kernel becomes more local, leading to greater curvature of the decision surface. When *σ* is small, the value of the discriminant function is nonzero only in the close vicinity of each SV, leading to a discriminant that is essentially constant outside the close proximity of the region where the data are concentrated (Figure 7C).

As seen from the examples in Figures 6 and 7, the width parameter of the Gaussian kernel and the degree of polynomial kernel determine the flexibility of the resulting SVM in fitting the data. Large degree or small width values can lead to overfitting and suboptimal performance (Figure 7C).

Results on a much larger sample of the two dimensional splice site recognition dataset are shown in Table 1. We observe that the use of a nonlinear kernel, either Gaussian or polynomial, leads to a small improvement in classifier performance when compared to the linear kernel. For the large degree polynomial and small width Gaussian kernel, we obtained reduced accuracy, which is the result of a kernel that is too flexible, as described above.

**Table 1:** SVM accuracy on the task of acceptor site recognition using polynomial and Gaussian kernels with different degrees *d* and widths *σ*.

Kernel	auROC
Linear	88.2%
Polynomial *d* = 3	91.4%
Polynomial *d* = 7	90.4%
Gaussian *σ* = 100	87.9%
Gaussian *σ* = 1	88.6%
Gaussian *σ* = 0.01	77.3%

Accuracy is measured using the area under the ROC curve (auROC) and is computed using 5-fold cross-validation (cf. the section Running Example: Splice Site Recognition for details).

#### Kernels for sequences

So far we have shown how SVMs perform on our splice site example if we use kernels based only on the two GC content features derived from the exonic and intronic parts of the sequence. The small subset of the dataset shown in Figures 1–7 seems to suggest that these features are sufficient to distinguish between the true splice sites and the decoys. This is not the case for a larger dataset, where examples from the two classes highly overlap. Therefore, to be able to separate true splice sites from decoys, one needs additional features derived from the same sequences. For instance, one may use the count of all four letters on the intronic and exonic part of the sequence (leading to eight features), or even all dimers (32 features), trimers (128 features), or longer ℓ-mers (2 · 4^ℓ^ features).

##### Kernels describing ℓ-mer content

The above idea is realized in the so-called *spectrum kernel* that was first proposed for classifying protein sequences [27],[28]:
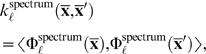
(11)where **x̅**,**x̅**′ are two sequences over an alphabet Σ, e.g., protein or DNA sequences. By |Σ|, we denote the number of letters in the alphabet. 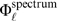 is a mapping of the sequence **x̅** into a |Σ|^ℓ^ dimensional feature space. Each dimension corresponds to one of the |Σ|^ℓ^ possible strings *s* of length ℓ and is the count of the number of occurrences of *s* in **x̅**. Please note that computing the spectrum kernel using the explicit computation of Φ will be inefficient for large ℓ: since it requires computation of the |Σ|^ℓ^ entries of the mapping Φ, which would be unfeasible for nucleotide sequences with ℓ≥10 or protein sequences with ℓ≥5. Faster computation is possible by exploiting the fact that the only ℓ-mers that contribute to the dot product (in Equation 11) are those that actually appear in the sequences. This leads to algorithms that are linear in the length of the sequences instead of the exponential |Σ|^ℓ^ computation time (see, e.g., [29] for more details and references).

If we use the spectrum kernel for the splice site recognition task, we obtain considerable improvement over the simple GC content features (see Table 2). The co-occurrence of long substrings is more informative than those of short ones. This explains the increase in performance of the spectrum kernel as the length of substrings ℓ is increased. Since the spectrum kernel allows no mismatches, when ℓ is sufficiently long the chance of observing common occurrences becomes small and the kernel will no longer perform well. This explains the decrease in the performance observed in Table 2 for ℓ = 5. This problem is alleviated if we use the *mixed spectrum kernel*:
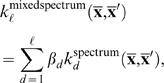
(12)where *β_d_* is a weighting for the different substring lengths (details below).

**Table 2:** The area under the ROC curve (auROC) of SVMs with the spectrum, mixed spectrum, and weighted degree kernels on the acceptor splice site recognition task for different substring lengths ℓ.

Kernel	auROC
Spectrum ℓ = 1	94.0%
Spectrum ℓ = 3	96.4%
Spectrum ℓ = 5	94.5%
Mixed spectrum ℓ = 1	94.0%
Mixed spectrum ℓ = 3	96.9%
Mixed spectrum ℓ = 5	97.2%
WD ℓ = 1	98.2%
WD ℓ = 3	98.7%
WD ℓ = 5	98.9%

##### Kernels using positional information

The kernels mentioned above ignore the position of substrings within the input sequence. However, in our example of splice site prediction, it is known that there exist sequence motifs near the splice site that allow the spliceosome to accurately recognize the splice sites. While the spectrum kernel is in principle able to recognize such motifs, it cannot distinguish where exactly the motif appears in the sequence. However, this is crucial in deciding where exactly the splice site is located. And indeed, Position Weight Matrices (PWMs) are able to predict splice sites with high accuracy. The kernel introduced next is analogous to PWMs in the way it uses positional information, and its use in conjunction with a large margin classifier leads to improved performance [30]. The idea is to analyze sequences of fixed length *L* and consider substrings starting at each position *l* = 1,…,*L separately*, as implemented by the so-called *weighted degree (WD) kernel*:
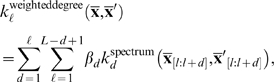
(13)where **x̅**_[*l*:*l*+*d*]_ is the substring of length *d* of **x̅** at position *l*. A suggested setting for *β_d_* is the weighting 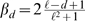 [29],[30]. Note that using the WD kernel is equivalent to using a mixed spectrum kernel for each position of the sequence separately (ignoring boundary effects). Observe in Table 2 that, as expected, the positional information considerably improves the SVM performance.

The *WD kernel with shifts* [31] is an extension of the WD kernel, allowing some positional flexibility of matching substrings. The locality improved kernel [32] and the *oligo kernel* [33] achieve a similar goal in a slightly different way.

Note that since the polynomial and Gaussian kernels are functions of the linear kernel, the above-described sequence kernels can be used in conjunction with the polynomial or Gaussian kernel to model more complex decision boundaries. For instance, the polynomial kernel of degree *d* combined with the ℓ-spectrum kernel, i.e.,
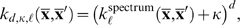
can model up to *d* co-occurrences of ℓ-mers (similarly proposed in [32]).

##### Other sequence kernels

Because of the importance of sequence data and the many ways of modeling it, there are many alternatives to the spectrum and weighted degree kernels. Most closely related to the spectrum kernel are extensions allowing for gaps or mismatches [28]. The feature space of the spectrum kernel and these related kernels is the set of *all* ℓ-mers of a given length. An alternative is to restrict attention to a predefined set of motifs [34],[35].

Sequence similarity has been studied extensively in the bioinformatics community, and local alignment algorithms like BLAST and Smith-Waterman are good at revealing regions of similarity between proteins and DNA sequences. The statistics produced by these algorithms do not satisfy the mathematical condition required of a kernel function. But they can still be used as a basis for highly effective kernels. The simplest way is to represent a sequence in terms of its BLAST/Smith-Waterman scores against a database of sequences [36]. This is a general method for using a similarity measure as a kernel. An alternative approach taken was to modify the Smith-Waterman algorithm to consider the space of *all* local alignments, leading to the *local alignment kernel* [37].

Probabilistic models, and Hidden Markov Models in particular, are in wide use for sequence analysis. The dependence of the log-likelihood of a sequence on the parameters of the model can be used to represent a variable-length sequence in a fixed dimensional vector space. The so-called Fisher-kernel uses the sensitivity of the log-likelihood of a sequence with respect to the model parameters as the feature space [38] (see also [39]). The intuition is that if we were to update the model to increase the likelihood of the data, this is the direction a gradient-based method would take. Thus, we are characterizing a sequence by its effect on the model. Other kernels based on probabilistic models include the Covariance kernel [40] and Marginalized kernels [41].

## Summary and Further Reading

This tutorial introduced the concepts of large margin classification as implemented by SVMs, an idea that is both intuitive and also supported by theoretical results in statistical learning theory. The SVM algorithm allows the use of kernels, which are efficient ways of computing scalar products in nonlinear feature spaces. The “kernel trick” is also applicable to other types of data, e.g., sequence data, which we illustrated in the problem of predicting splice sites in *C. elegans*.

In the rest of this section, we outline issues that we have not covered in this tutorial and provide pointers for further reading. For a comprehensive discussion of SVMs and kernel methods, we refer the reader to recent books on the subject [2],[5],[7].

### 

#### Normalization

Large margin classifiers are known to be sensitive to the way features are scaled (see, for example [42], in the context of SVMs). It can therefore be essential to normalize the data. This observation carries over to kernel-based classifiers that use nonlinear kernel functions. Normalization can be performed at the level of the input features or at the level of the kernel (normalization in feature space), or both. When features are measured in different scales and have different ranges of possible values, it is often beneficial to scale them to a common range, e.g., by *standardizing* the data (for each feature, subtracting its mean and dividing by its standard deviation). An alternative to normalizing each feature separately is to normalize each example to be a unit vector. This can be done at the level of the input features by dividing each example by its norm, i.e., **x̃**: = **x**/∥**x**∥, or at the level of the kernel which normalizes in the feature-space of the kernel, i.e., 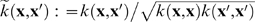. For the discussed splice site data, the results differed considerably when using different normalizations for the linear, polynomial, and Gaussian kernels. Generally, our experience shows that normalization often leads to improved performance for both linear and nonlinear kernels, and can also lead to faster convergence.

#### Handling unbalanced data

Many datasets encountered in bioinformatics and other areas of application are unbalanced, i.e., one class contains a lot more examples than the other. For instance, in the case of splice site detection, there are 100 times fewer positive examples than negative ones. Unbalanced datasets can present a challenge when training a classifier, and SVMs are no exception. The standard approach to addressing this issue is to assign a different misclassification cost to each class. For SVMs, this is achieved by associating a different soft-margin constant to each class according to the number of examples in the class (see, e.g., [43] for a general overview of the issue). For instance, for the splice site recognition example, one may use a value of *C* (in Equation 3) that is 100 times larger for the positive class than for the negative class. Often when data is unbalanced, the cost of misclassification is also unbalanced; for example, having a false negative is more costly than a false positive. In some cases, considering the SVM score directly rather than just the sign of the score is more useful.

#### Kernel choice and model selection

A question frequently posed by practitioners is “which kernel with which parameters should I use for my data?” There are several answers to this question. The first is that it is, like most practical questions in machine learning, data-dependent, so several kernels should be tried. That being said, one typically follows the following procedure: try a linear kernel first, and then see if we can improve on its performance using a nonlinear kernel. The linear kernel provides a useful baseline, and in many bioinformatics applications it is hard to beat, in particular if the dimensionality of the inputs is large and the number of examples small. The flexibility of the Gaussian and polynomial kernels can lead to overfitting in high-dimensional datasets with a small number of examples, such as in micro-array datasets. If the examples are (biological) sequences, then the spectrum or the WD kernel of relatively low order (say ℓ = 3) are good starting points if the sequences have varying or fixed length. Depending on the problem, one may then try the spectrum kernel with mismatches, the oligo kernel, the WD kernel with shifts, or the local alignment kernel.

In problems such as prediction of protein function or protein interactions, there are several sources of genomic data that are relevant, each of which may require a different kernel to model. Rather than choosing a single kernel, several papers have established that using a combination of multiple kernels can significantly boost classifier performance [44]–[46].

When selecting the kernel, its parameters, and the soft-margin parameter *C*, one has to take care that this choice is made completely independently of the examples used for performance evaluation of the method. Otherwise, one will overestimate the accuracy of the classifier on unseen data points. This can be done by suitably splitting the data into several parts, where one part, say 50%, is used for training, another part (20%) for tuning of SVM and kernel parameters, and a third part (30%) for final evaluation. Techniques such as *N*-fold cross-validation can help if the parts become too small to reliably measure prediction performance (see, for example, [47],[48]).

#### Kernels for other data types

We have focused on kernels for real-valued and sequence data; and while this covers many bioinformatics applications, often data is better modeled by more complex data types. Many types of bioinformatics data can be modeled as graphs, and the inputs can be either nodes in the graph, e.g., proteins in an interaction network, or the inputs can be represented by graphs, e.g., proteins modeled by phylogenetic trees. Kernels have been developed for both scenarios. Researchers have developed kernels to compare phylogenetic profiles modeled as trees [49], protein structures modeled as graphs of secondary-structural elements [50],[51], and graphs representing small molecules [52]. The diffusion kernel is a general method for propagating kernel values on a graph [53]. Several of the kernels described above are based on the framework of *convolution kernels* [54], which is a method for developing kernels for an object based on kernels defined on its sub-parts, such as a protein structure composed of secondary structural elements [50]. Kernels (and hence the similarity) on structured data can also be understood as how much one object has to be transformed before it is identical to the other, which leads to the idea of transducers [55]. More details on kernels can be found in books such as [2],[5],[7],[56].

#### SVM training algorithms and software

The popularity of SVMs has led to the development of a large number of special-purpose solvers for the SVM optimization problem [57]. *LIBSVM* [42] and *SVM^light^* [58] are two popular examples of this class of software. The complexity of training of nonlinear SVMs with solvers such as *LIBSVM* has been estimated to be quadratic in the number of training examples [57], which can be prohibitive for datasets with hundreds of thousands of examples. Researchers have therefore explored ways to achieve faster training times. For linear SVMs, very efficient solvers are available that converge in a time that is linear in the number of examples [57],[59],[60]. Approximate solvers that can be trained in linear time without a significant loss of accuracy were also developed [61].

Another class of software includes machine learning libraries that provide a variety of classification methods and other facilities such as methods for feature selection, preprocessing, etc. The user has a large number of choices, and the following is an incomplete list of environments that provide an SVM classifier: *Orange* [62], *The Spider* [63], *Elefant* [64], *Plearn* [65], *Weka* [66], Lush [67], *Shogun* [68], *RapidMiner* [69], *PyML* [70], and *Easysvm* [17]. The SVM implementations in several of these packages are wrappers for the *LIBSVM* [42] or *SVM^light^* [58] library. The *Shogun* toolbox contains eight different SVM implementations together with a large collection of different kernels for real-valued and sequence data.

A repository of machine learning open source software is available at http://mloss.org as part of an initiative advocating distribution of machine learning algorithms as open source software [71].
